# Urban-Rural Disparities in Dental Services Utilization Among Adults in China's Megacities

**DOI:** 10.3389/froh.2021.673296

**Published:** 2021-06-09

**Authors:** Xiang Qi, Xiaomin Qu, Bei Wu

**Affiliations:** ^1^Rory Meyers College of Nursing, New York University, New York, NY, United States; ^2^School of Social Development, East China University of Political Science and Law, Shanghai, China

**Keywords:** urban-rural, oral health, dental care use, dental visit, Chinese

## Abstract

**Objective:** China's dental care system is bifurcated between urban and rural areas. However, very few studies have examined the dental services utilization inequities in China's megacities, particularly in these urban and rural areas. This study aims to examine the urban-rural disparities in dental services utilization among adults living in China's megacities based on the Andersen dental services utilization model.

**Methods:** This study used data from 4,049 residents aged 18–65 who participated in the “2019 New Era and Living Conditions in Megacities Survey.” Multivariate logistic regressions were employed to examine the associations between place of residence and dental services utilization for individuals from ten megacities in China. Predisposing variables (age, gender, marital status, living arrangement, and education), enabling variables (socioeconomic status, occupational status, income, insurance coverage, health attitude, and health behavior), and need variables (self-rated health, oral health status, gum bleeding) were controlled for.

**Results:** The mean age of the 4,049 adults was 45.2 (standard deviation = 13.0), and 30.4% (*n* = 1,232) had no dental visits at all. Adults who resided in urban areas were more likely to use dental services [odds ratio (OR) = 1.57, 95% confidence interval (CI) = 1.30 to 1.91] than those residing in rural areas after controlling for key covariates. Factors associated with higher odds of visiting dentists include having a higher income (OR = 1.44, *P* < 0.001), higher education level (OR = 1.53, *P* = 0.042), being covered by insurance for urban residents/employees (OR = 1.49, *P* = 0.031), having a positive attitude toward healthy diets (OR = 1.43, *P* < 0.001), attending regular physical examination (OR = 1.66, *P* < 0.001), having more tooth loss (OR = 1.05, *P* < 0.001), and having frequent gum bleeding (OR = 2.29, *P* < 0.001).

**Conclusion:** The findings confirm that place of residence is associated with dental services utilization while adjusting for key covariates. Despite rapid economic development in China, many adults had never visited dentists at all. More efforts should be taken to encourage widespread dental care, such as providing more dental coverage and better access to dental care services.

## Introduction

Oral health problems and disorders are common among adults in the Chinese population. According to the 4th National Oral Health Epidemiology Survey conducted between 2015 and 2016 in China, the prevalence of dental caries is more than 90.0% for adults between the ages of 35 and 74 years old [1]. Furthermore, the prevalence of periodontitis among middle-aged adults ranging from 35 to 44 years old is 52.8% [2]. Such oral health diseases and problems are associated with a lack of dental services utilization [3–5]. Moreover, the use of dental care services among the Chinese population is very low compared to those in developed countries [6–10]. Due to its direct association with the persistence of oral health issues, it is important to identify factors associated with dental services utilization in China. The knowledge generated in this area would provide a better understanding of service utilization patterns, assist in designing a cost-effective dental care system and promote policy change in the future.

Despite China's significant economic development in the past three decades, large disparities in income, infrastructure, and social services between urban and rural areas remain. For example, the ratio of urban to rural income was 1.86 in 1985, and it increased to 2.56 in 2019 [11]. According to the Chinese Health Statistics Yearbook of 2018, urban areas had 535 hospitals specializing in dental treatments in 2017; in contrast, rural areas only had 154 dental hospitals. People living in urban areas are more likely to have higher levels of income and education than their rural counterparts, as well as have retirement pensions [12, 13]. Additionally, rural residents encounter barriers in obtaining basic public services and welfare, such as the healthcare system and social security coverage [14]. Such urban-rural disparities impose a significant barrier that hinders individuals residing in rural areas from accessing dental care; studies have generally reported a lower use of dental care services in rural areas [8, 15–17]. Due to the unbalanced development across urban and rural areas, dental health resources, including the dental department and dental workforce, are insufficient and distributed unevenly between the two areas [18]. Although there are no national statistics on the dental care expenses in China, some estimates suggest that 85% of dental care cost is paid out-of-pocket [18, 19]. Rural residents are more likely to postpone dental appointments due to the inaccessibility of dental services and their inability to pay out-of-pocket dental care expenses [7].

The urban-rural divide also exists in megacities that exhibit a higher development level than other areas of China [20]. A megacity is defined as containing over five million residents [21], and under this definition there were 16 megacities in China in 2019 [22]. Unlike developed countries, all of China's megacities are metropolitan regions that include urban, peri-urban and rural land, and all have rural populations within the city boundaries. Because their development level is higher than other parts of China, the urban-rural divide in China's megacities has significantly narrowed with respect to income, education level, housing, and social welfare programs since 1990 [20, 23, 24]. However, the reduced urban-rural income divide does not significantly diminish the urban-rural disparities in healthcare service allocation and distribution [25]. Understanding the dental services utilization in China's megacities is of particular importance because of their mega-sized populations, rapid economic development, and millions of rural migrants without urban household registration status [20]. Nonetheless, very few studies have been conducted on dental services utilization among adults in China's megacities.

This study aimed to address the knowledge gap by examining how dental service utilization was associated with place of residence among adult populations in China's megacities. Based on existing literature on dental services use [6–10], we hypothesized that dental services utilization would be associated with urban area residence after controlling for key covariates.

## Methods

### Samples and Data Collection

This study used the data from the “2019 New Era and Living Conditions in Megacities Survey (NELCMS).” This survey is a cross-sectional observational study using a multi-stage, stratified sampling design and focuses on policy issues related to social structure and social mobility among adults in China's megacities. It was conducted in ten megacities in China, including the most economically developed megacities characterized by a Gross Domestic Product (GDP) per capita higher than 134,000 RMB (equivalent to 19,619 U.S. dollars). Namely, the megacities surveyed were Beijing, Shanghai, Guangzhou, Shenzhen, Tianjin, Hangzhou, Chongqing, Chengdu, Wuhan, and Changsha. The questionnaire consists of two volumes, including 418 items. The main contents included the following aspects: demographic characteristics, work and social security, socioeconomic status, living and household conditions, activities of daily living assessment, and health status and behavior habits.

Using the probability-proportional-to-size sampling method, researchers selected participants through four stages: city, community, household, and individual. Forty communities were randomly selected from each city. Twenty-five households were randomly selected from the housing registration database obtained from each community. One individual was randomly selected from each household. The study was approved by the Ethics Committee of Shanghai University (ECSHU 2020–096).

Inclusion criteria for participation were: (a) self-identified as Chinese and able to communicate in Mandarin; (b) full-time residence in this city for more than 6 months in the past year; (c) age between 18 and 65; (d) capable of answering interview questions. In this survey, questionnaires included Volume A and Volume B. Volume A is the core interview that focuses on social mobility and social structure. Volume B covers individuals' health-related questions, such as health status, health insurance, and health care utilization. Participants were randomly selected to complete either Volume A or Volume B. To address the study aim, only participants who completed Volume B were selected.

In full accordance with ethical principles, well-trained interviewers collected the data during in-home interviews in Mandarin. The informed consent was obtained prior to the data collection. The interviews lasted about 45–60 min. To ensure that the questionnaire was reliable and valid, we conducted a pilot study on 50 adults. Based on their feedback and suggestions, the questionnaire was revised and finalized before the formal data collection. From July 2019 to August 2019, the research team collected data from a total of 5,000 participants who filled out Volume B of the questionnaire. After excluding 815 participants with missing values on the individual's and father's International Socio-Economic Index of Occupational Status (ISEI) and 136 participants with missing values on the study variables, the final analytical sample consisted of 4,049 respondents.

### Measures

#### Dependent and Independent Variables

The dependent variable in our analysis is dental services utilization. The survey asked, “how often do you visit a dentist for dental care?” The responses are the frequency of dental visits (0 = never, 1 = rarely, 2 = less than once every two years, 3 = at least once every 2 years, 4 = at least once a year, 5 = twice a year). Dental care utilization in China is at a very low level; a national survey in 2015–2016 found that 78.6% of adults aged 35–44 and 79.3% of older adults aged 65–74 had not used dental care services in the past 12 months [17]. We therefore dichotomized the responses into 1 = “never” and 0 = “otherwise.” The independent variable, place of residence, was dichotomized into 1 = urban or 0 = rural.

#### Covariates

**Figure 1:**
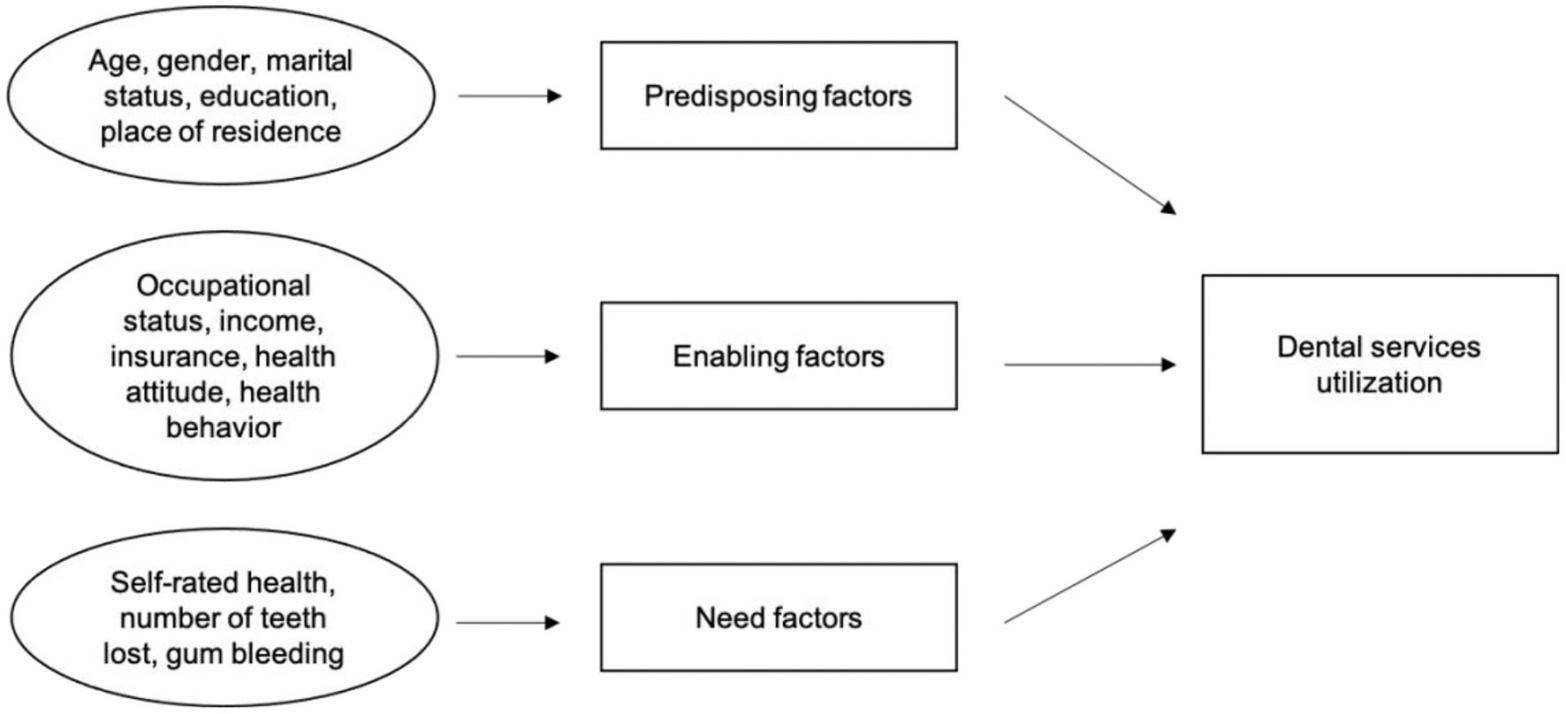
Modified andersen dental services utilization model.

This study applies the Andersen Healthcare Utilization Model (Figure 1) [26, 27] to guide the selection of variables. According to this model, people's use of dental services over a given period is a function of predisposing factors, enabling factors, and need factors.

##### Predisposing Factors

Demographic information includes age (18–25, 26–30,., 61–65 years old, nine categories ranging from 18 to 65), gender (0 = female, 1 = male), marital status (1 = married or living with partner, 0 = otherwise), and education (0 = illiterate/elementary school, 1 = middle/high/vocational school, 3 = 3 years college or more).

##### Enabling Factors

Early-life and current socioeconomic status include the father's and individual's occupational status determined by the International Socio-Economic Index of Occupational Status (ISEI) developed by Ganzeboom et. al. [28], and individual's annual income (0 = < 78,000 RMB, 1 = ≥ 78,000 RMB). To facilitate interpretation of findings, ISEI were dichotomized into 2 groups [0 = < 54 (low), 1 = ≥ 54 (high)]. Health insurance coverage consists of 0 = no health insurance, 1 = New Cooperative Medical Scheme for rural residents, 2 = Urban Residents Basic Medical Insurance, 3 = Urban Employees Basic Medical Insurance, and 4 = other insurance (mostly commercial insurance). In addition, we measured the respondents' health attitude by asking whether they are concerned about eating a healthy diet. The response was coded as 0 = no, and 1 = yes. Health behaviors were measured by the regularity of attending physical exams (having physical exam once a year, 0 = no, 1 = yes).

##### Need Factors

Self-rated health was measured by nine questions acquired from the Self-Rated Health Measurement Scale [29]. Each item was scored on a scale of 0–10, with higher values representing better self-rated health. The potential range of the scale is 0 to 90. The Cronbach's alpha for the measure was 0.833, which showed a high internal consistency. Oral health status was determined by self-reported tooth loss (the number of missing teeth), and self-reported gum bleeding (0 = never/hardly, 1 = occasionally/frequently/always).

### Statistical Analysis

**Figure 2:**
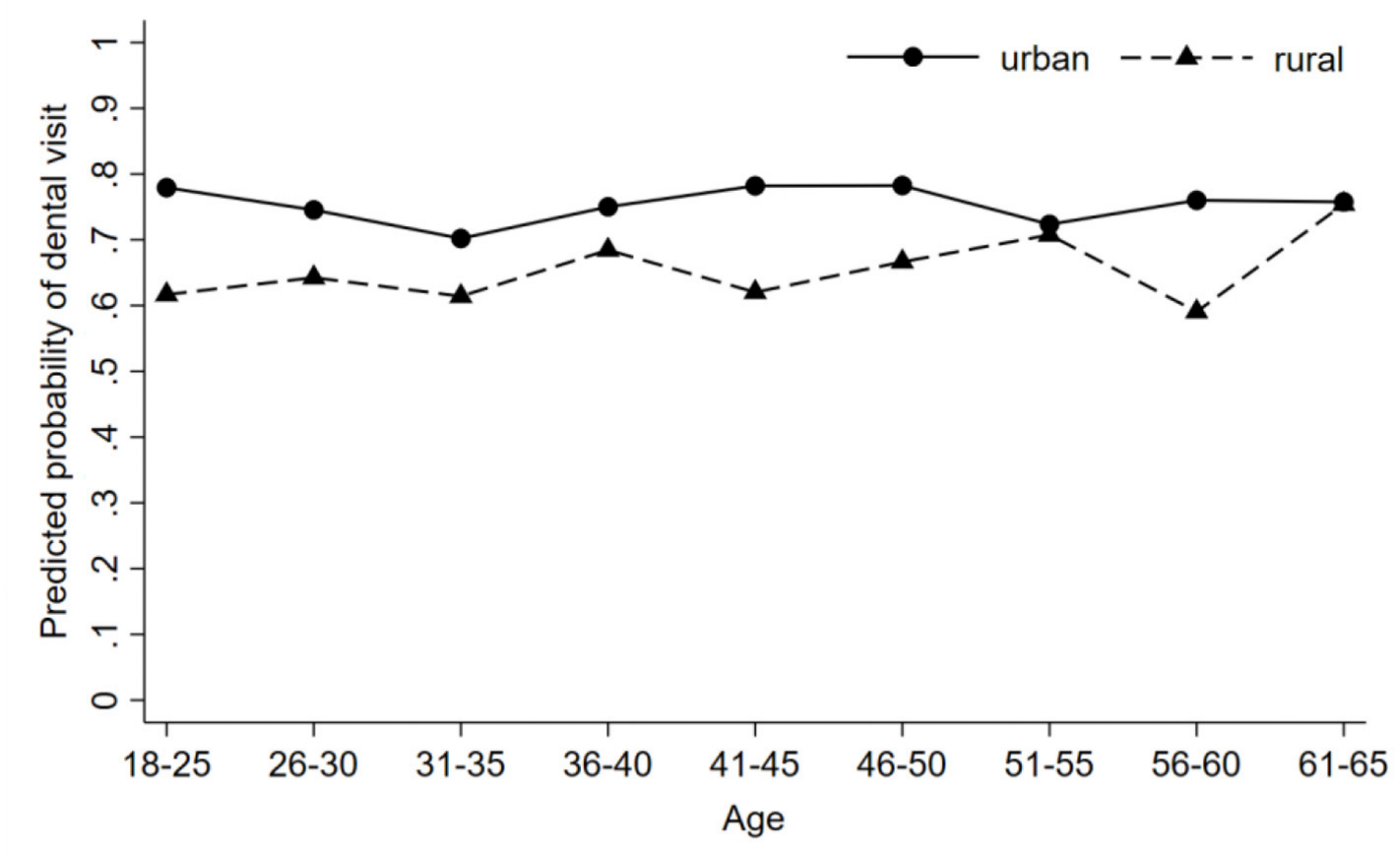
Disparities in predicted probability of having dental visit by place of residence (urban vs. rural) with 95% confidence intervals (*N* = 4,049, weighted). All estimations include gender, marital status, education, attending the regular physical exam, care about eating a healthy diet, father's occupational status, individual's occupational status, income, insurance, self-rated health, tooth loss, and gum bleeding as covariates.

Post-stratification individual-level sampling weights were used to adjust for differences in the individual-by-household-by-community-by-city distribution between the sample and the general population in the ten megacities in the study [30]. We defined city as sampling unit (*n* = 10), and neighborhood as strata (*n* = 40). All analyses presented in Tables 1, 2 and Figure 2 were adjusted for sampling weight.

**Table 1:** Sample characteristics (*N* = 4,049, weighted).

	**Total sample**	**Residents with dental visits (*****N*** **=** **2,817)**		**Residents with no dental visits (*****N*** **=** **1,232)**	
	***N* = 4,049**	**Urban (*N* = 2,037)**	**Rural (*N* = 780)**	**Urban (*N* = 637)**	**Rural (*N* = 595)**
	**%/Mean (SD)**	**%/Mean (SD)**	**%/Mean (SD)**	**%/Mean (SD)**	**%/Mean (SD)**
**PREDISPOSING VARIABLES**					
**Gender**					
Male	46.6	44.1	46.0	51.8	50.3
**Age**					
18–25	6.3	5.2	8.6	4.4	9.1
26–30	10.7	9.6	14.3	8.3	12.3
31–35	11.8	10.9	12.9	12.4	12.9
36–40	10.8	11.3	11.0	10.6	8.8
41–45	10.3	10.3	10.1	9.0	12.4
46–50	12.0	11.9	12.0	10.7	13.8
51–55	9.7	8.3	10.9	10.6	11.8
56–60	10.8	11.9	7.1	12.5	9.7
61–65	17.6	20.6	13.1	21.5	9.2
**Marital status**					
Married/living with partner	80.1	79.6	79.7	79.4	83.3
**Education**					
Illiterate/elementary school	8.7	3.7	14.3	6.4	20.6
Middle/high/vocational school	50.6	43.9	56.1	54.6	62.1
3 years college or more	40.7	52.4	29.6	39.0	17.3
**ENABLING VARIABLES**					
**Father's occupational status (ISEI)**					
High (≥54)	31.1	40.3	16.8	36.3	13.1
**Individual's occupational status (ISEI)**					
High (≥54)	33.3	44.2	24.2	28.4	13.4
**Income**					
High (≥78,000)	32.2	37.9	31.6	29.7	16.1
**Insurance**					
No health insurance	8.5	5.6	10.2	10.4	14.0
New Cooperative Medical Scheme	11.1	1.2	25.4	2.2	35.4
Urban Resident Basic Medical Insurance	22.0	23.9	18.9	24.6	16.8
Urban Employee Basic Medical Insurance	51.7	60.6	40.7	56.2	30.8
Other medical insurance	6.7	8.7	4.8	6.6	3.0
**Regular physical exam**					
Yes	60.7	72.5	54.5	55.0	34.4
**Care about eating a healthy diet**					
Yes	56.9	64.5	54.9	50.9	40.0
**NEED VARIABLES**					
**Self-rated health**					
SRHMS (range:0–90)	61.5 (12.7)	61.0 (12.4)	61.2 (12.4)	61.8 (13.2)	63.1 (13.2)
**Tooth loss**					
Number of missing teeth (range:0-28)	1.5 (3.3)	1.7 (3.4)	1.5 (2.8)	1.3 (3.4)	1.0 (3.0)
**Gum bleeding**					
Yes (occasionally/frequently/always)	50.9	56.1	59.1	37.4	36.6

*SD, standard deviation; ISEI, international socio-economic index of occupational status; SRHMS, self-rated health measurement scale*.

**Table 2:** Multivariate logistic regression models of having dental services utilization at least once (*N* =4,049, weighted).

	**Model 1**		**Model 2**		**Model 3**		**Model 4**	
	**OR**	**95% CI**	**OR**	**95% CI**	**OR**	**95% CI**	**OR**	**95% CI**
**Place of residence (Ref. Rural)**								
Urban	2.442^***^	2.095–2.845	1.731^***^	1.460–2.052	1.566^***^	1.299–1.889	1.573^***^	1.296–1.908
**PREDISPOSING VARIABLES**								
**Gender (Ref. Female)**								
Male			0.810^**^	0.691–0.949	0.773^**^	0.657–0.909	0.777^**^	0.658–0.918
**Age (Ref. 18–25)**								
26–30			1.079	0.726–1.603	1.006	0.680–1.488	0.976	0.658–1.448
31–35			0.909	0.604–1.367	0.855	0.571–1.281	0.819	0.545–1.230
36–40			1.232	0.812–1.871	1.160	0.767–1.756	1.068	0.702–1.625
41–45			1.196	0.780–1.833	1.101	0.719–1.684	1.053	0.688–1.611
46–50			1.359	0.891–2.072	1.270	0.837–1.927	1.146	0.752–1.745
51–55			1.243	0.797–1.936	1.209	0.780–1.873	1.044	0.669–1.631
56–60			1.206	0.784–1.855	1.181	0.771–1.807	0.969	0.627–1.498
61–65			1.504	0.992–2.279	1.485	0.986–2.236	1.185	0.777–1.806
**Marital status (Ref. Otherwise)**								
Married/living with partner			0.861	0.686–1.081	0.848	0.676–1.064	0.859	0.682–1.081
**Education (Ref. Illiterate/elementary school)**								
Middle/high/vocational school			1.301	0.988–1.713	1.203	0.910–1.590	1.225	0.913–1.643
3 years college or more			2.165^***^	1.567–2.993	1.537^*^	1.082–2.182	1.529^*^	1.061–2.205
**ENABLING VARIABLES**								
**Father's ISEI (Ref. Low)**								
High					1.010	0.844–1.209	1.014	0.844–1.218
**Individual's ISEI (Ref. Low)**								
High					1.422^***^	1.167–1.732	1.490^***^	1.222–1.817
**Income (Ref. Low)**								
High					1.382^**^	1.131–1.689	1.438^***^	1.175–1.760
**Insurance (Ref. No health insurance)**								
New Cooperative Medical Scheme					1.070	0.765–1.498	1.098	0.775–1.554
Urban Resident Basic Medical Insurance					1.474^*^	1.094–1.986	1.570^**^	1.154–2.135
Urban Employee Basic Medical Insurance					1.335^*^	1.011–1.763	1.383^*^	1.040–1.838
Other medical insurance					1.608^*^	1.053–2.455	1.763^*^	1.147–2.708
**Regular physical exam (Ref. Low)**								
Yes					1.669^***^	1.410–1.977	1.661^***^	1.399–1.972
**Care about eating a healthy diet (Ref. No)**								
Yes					1.457^***^	1.238–1.716	1.429^***^	1.209–1.689
**NEED VARIABLES**								
Self–rated health (SRHMS)							0.901	0.766–1.059
Tooth loss (Number of missing teeth)							1.053^**^	1.017–1.092
**Gum bleeding (Ref. No)**								
Yes (occasionally/frequently/always)							2.286^***^	1.948–2.682

*OR, odds ratio; CI, confidence interval. Significance level*:

* *p < 0.05*,

** *p < 0.01*,

*** *p < 0.001. ISEI, international socio-economic index of occupational status; SRHMS, self-rated health measurement scale*.

We first used descriptive statistics, including proportions and 95% confidence intervals (CIs), to consider the complex sampling and sampling weights. Bivariate analyses were conducted using Chi-Square tests to estimate urban-rural disparities in dental visits. Multivariate stepwise logistic regressions were conducted, according to the Andersen Healthcare Utilization Model [27] and previous literature [16, 31]. We only included the place of residence in the first step (Model 1). In the second step, we entered predisposing factors, including age, gender, marital status, and education. Next, we added enabling factors, which consisted of the father's and individual's occupational class, income, insurance, health attitude, and health behavior (Model 3). In the final Model 4, we added need factors, including self-rated health and oral health status of the participants.

We used margins post estimation to examine whether the urban-rural disparities in dental services utilization were associated with a change in age after controlling for other covariates [32, 33]. We used STATA (Version 15.0) for all statistical analyses. A *P*-value ≤ 0.05 was considered significant.

## Results

The sample characteristics are summarized in Table 1. In the weighted analytical sample, 28.23% of urban residents and 17.58 % of rural residents had at least one dental visit per year. In addition, 637 (23.82%) urban residents and 595 (43.27%) rural residents never had any dental visits.

Bivariate analyses did not find urban-rural differences in the distributions of gender, age, and marital status. However, in comparison to residents in urban areas, those in rural areas were less likely to have dental visits [76.18% (urban) vs. 56.73% (rural)]. Rural residents had lower income, lower levels of education, and lower levels of father's and individual's occupational status. Additionally, rural residents were less concerned about eating a healthy diet and less likely to have regular physical exams compared with their urban counterparts. No significant urban-rural difference was found in self-rated health and oral health status.

Table 2 presents the results from the stepwise logistic regression models on visiting a dentist for Chinese adults in megacities. Place of residence had a significant association with dental visits in Model 1 (including the variable on place of residence only) [Odds Ratio (OR) = 2.44, 95% CI = 2.10 to 2.85].

In Model 2, male adults were less likely to visit a dentist (OR = 0.81, 95% CI = 0.69 to 0.95). Education beyond three-year college was significantly associated with dental visits (OR = 2.17, 95% CI = 1.57 to 2.99).

Individuals with a higher level of socioeconomic status (i.e., education, income, and occupational status) were more likely to visit a dentist (*P* < 0.05) (Model 3). Additionally, in comparison with those who did not have insurance, residents with the New Cooperative Medical Scheme (health insurance for rural residents) had similar odds of visiting a dentist. Individuals who were more concerned about a healthy diet and had regular physical examinations were more likely to visit a dentist.

Among need variables, self-rated health was not related to dental visits. In the fully specified model (Model 4), the odds of visiting a dentist were 57.3% higher for urban residents than their rural counterparts (OR = 1.57, 95% CI = 1.30 to 1.91). Moreover, individuals with fewer remaining teeth (OR = 1.05, 95% CI = 1.02 to 1.09) and gum bleeding symptoms were more likely to visit a dentist (OR = 2.29, 95% CI = 1.95 to 2.68).

Adjusting for other covariates (demographics, socioeconomic status, health attitude and behavior, self-rated health, and oral health status), Figure 2 shows the margins post estimation of predicted probability of visiting a dentist across age groups and by place of residence. Overall, rural residents were less likely to use dental care compared with their urban counterparts in all age groups.

## Discussion

This study has provided a better understanding of dental services utilization in megacities by demonstrating that place of residence was associated with dental services utilization while adjusting for key covariates. Data from ten megacities in China were employed, and the individuals' socioeconomic conditions, health insurance, health attitude and behaviors, and oral health status were found to be significant explanatory variables. In addition, the urban-rural disparities in dental services utilization remained regardless of age.

Never visiting a dentist is a common phenomenon among the adult population in China. Our study showed that 30.4% of the respondents had never experienced a dental visit. Furthermore, a study conducted in China using national data found the rates of not visiting a dentist in the past 12 months were relatively high-−78.6% for adults aged 35 to 44, and 79.3% for older adults aged 65 to 74 [17]. According to the data from the National Health and Nutrition Examination Survey (NHANES) conducted in the U.S. from 2011 to 2014, 33.8% of adults aged over 30 reported not having a dental visit in the past 12 months [6]. Our study shows that even in these highly economically developed megacities in China, the rate of dental visits is much lower than those in developed or high-income countries [9, 10].

Urban-rural disparities are reflected by the predisposing factors. Affordability of dental service could be another significant reason for the limited dental visits, particularly among residents from rural areas. This is consistent with a study conducted in 14 European countries among older adults that found older adults with lower income were less likely to seek dental care [9]. Our study found that the type of health insurance possessed by an individual strongly associated with dental services utilization. Although the Chinese government has provided universal basic medical coverage since 2009 [34], no health insurance covers the expenses for preventative oral health services in China. There are still various out-of-pocket costs for dental visits, depending on the type of insurance. Compared with the Urban Resident Basic Medical Insurance (URBMI) and Urban Employee Basic Medical Insurance (UEBMI) for urban residents, the New Cooperative Medical Scheme (NCMS) for rural residents has less medical coverage. The NCMS also has complicated and ambiguous reimbursement procedures [14, 34, 35]. The NCSM covers only partial medical expenses and no dental treatment expenses [36]. This can be reflected in our study, as having NCSM did not increase the odds for dental visits. Thus, expanding dental services with a lower amount of out-of-pocket cost for adults could be a strategy to increase dental services utilization.

Within the enabling factors domain, the present study found that concerning about eating a healthy diet was significantly related to dental services utilization, consistent with previous studies [16, 31]. The Chinese population has a long tradition of attributing oral diseases to the food taken [37]. Oral health literacy has an important role in addressing oral health problems because it is associated with oral health behaviors (e.g., use of dental floss, regular toothbrushing) [38] and oral health-seeking behavior [39]. Similarly, having regular physical exams can reflect individuals' health literacy and health conscientiousness [40]. Adults living in urban areas have easier access to regular physical exams and are more likely to seek dental care. Studies have also indicated that oral health belief was associated with increased dental service use [4, 37]. Although a “National Love your Teeth Day” (September 20th) has been designated by the Chinese government to increase public awareness of oral health since 1989 [41], additional efforts are warranted to enhance public knowledge of oral diseases and problems and the importance of preventive dental visits.

Regarding the need factors for dental care, studies conducted in developed countries revealed that compromised oral health status was negatively associated with preventive dental services utilization [9]. Opposite from these previous findings, our study found that poorer oral health was positively associated with dental visits in China's megacities. Presumably, this contradictory finding could be attributed to the different approaches China and developed countries take in addressing oral health. Chinese dental visits are treatment-oriented and driven by severe dental symptoms, which means that people do not seek dental care unless they feel unbearable toothaches or experience severe periodontal symptoms [7]. On the other hand, in developed countries it is common to have regular dental visits that are prevention-oriented, such as tooth cleaning, dental check-ups, or examination [6]. Another factor could be that cultural attitudes toward oral health are different. Wu et al. [42] suggested that perceptions of oral health and oral health beliefs are embedded in social and cultural contexts. Most Chinese people tend to utilize traditional remedies such as drinking green tea and taking Vitamin C rather than seeking professional dental services for treatments [43].

The strengths of this study reside in its focus on the comparisons between urban and rural residents in China's megacities. This is one of the first studies devoted to revealing urban-rural disparities in dental services utilization among adult populations in China's megacities. This study is also unique in investigating whether the urban-rural disparities vary by age. In China, very few studies have been conducted on inequalities in dental services utilization. Moreover, the data were collected from ten megacities that can represent many regions of the urban areas in China.

There are a few limitations in this study that need to be acknowledged. First, this survey does not contain information on reasons for dental visits (preventive, treatment, or emergency visits), nor the time frame of each individual's dental visits. Second, respondents' health attitude was measured by asking whether they are concerned about eating a healthy diet. The absence of relevant information on oral health attitude is a limitation. Third, given the nature of the cross-sectional data, the possibilities of establishing a causal relationship between dental service utilization and the explanatory variables are precluded. Fourth, data in this study were derived from respondents' self-reports, which may lead to recall bias. Future studies should consider using objective measures of clinical oral examinations and including other factors that might influence an individual's dental services utilization, such as oral health literacy, personal health choices, and psychosocial factors. Furthermore, intervention strategies and related dental policies are warranted to ensure equitable access to and quality of dental services utilization in China.

## Conclusions

This study has extended literature by showing that place of residence is associated with dental services utilization while controlling for key confounding factors using data from ten megacities in China. Despite rapid economic development in China, some adults had never visited a dentist, even in the most economically developed megacities. More efforts should be implemented to improve dental care use by providing more dental care coverage and better access to dental care services.

## Data Availability Statement

The raw data supporting the conclusions of this article will be made available by the authors, without undue reservation.

## Ethics Statement

The studies involving human participants were reviewed and approved by Ethics Committee of Shanghai University (ECSHU 2020-096). The patients/participants provided their written informed consent to participate in this study.

## Author Contributions

XgQ contributed to the acquisition, analysis and interpretation of data, and draft of the article. XnQ contributed to the acquisition, analysis and interpretation of data and draft of the article. BW contributed to the conceptualization and design of the study, interpretation of the results, and draft of the article. All authors contributed to the article and approved the submitted version.

## Conflict of Interest

The authors declare that the research was conducted in the absence of any commercial or financial relationships that could be construed as a potential conflict of interest.
